# Increased expression of ATase1/NAT8B or ATase2/NAT8 in the mouse results in an autistic-like phenotype with altered dendritic branching and spine formation

**DOI:** 10.1038/s41380-025-03228-1

**Published:** 2025-09-24

**Authors:** Balagangadharan Kalimuthu, Haiyan Lu, Angelique Steenhagen, Qiping Dong, Mitchell Gray, Michael J. Rigby, Andreas Endresen, Qiang Chang, Lingjun Li, Luigi Puglielli

**Affiliations:** 1 Department of Medicine, University of Wisconsin-Madison, Madison, WI USA; 2 Waisman Center, University of Wisconsin-Madison, Madison, WI USA; 3 School of Pharmacy, University of Wisconsin-Madison, Madison, WI USA; 4 Department of Medical Genetics and Neurology, University of Wisconsin-Madison, Madison, WI USA; 5 Department of Chemistry, University of Wisconsin-Madison, Madison, WI USA; 6 Geriatric Research Education Clinical Center, Veterans Affairs Medical Center, Madison, WI USA; 7 Department of Neuroscience, University of Wisconsin-Madison, Madison, WI USA; 8 Present Address: National University of Singapore, Singapore, Singapore; 9 Present Address: Department of Neurology, Mayo Clinic, Rochester, MN USA; 10 Present Address: Boehringer-Ingelheim, Ridgefield, CT USA

**Keywords:** Neuroscience, Psychology

## Abstract

Neurons heavily depend on the ability of the secretory pathway to deliver correctly folded polypeptides to the periphery of the cell for the assembly, maintenance, and normal functioning of synapses. The endoplasmic reticulum (ER) acetylation machinery has emerged as a novel branch of the more general ER quality control machinery. It regulates the positive selection of correctly folded nascent glycoproteins, thus ensuring the efficiency of the conventional secretory pathway. ER acetylation requires the activity of two ER-luminal acetylCoA:lysine acetyltransferases, ATase1/NAT8B and ATase2/NAT8. Both acetyltransferases depend on the influx of acetyl-CoA into the ER from the cytosol, which is ensured by the coordinated action of the citrate transporters, SLC25A1 and SLC13A5, and the ER acetyl-CoA transporter, AT-1. Gene duplication events affecting ATase1 and ATase2 are associated with rare disease phenotypes that include autism and intellectual disability with dysmorphism. Here, we generated mice with neuron-specific overexpression of human ATase1 or ATase2. The animals display autistic-like behaviors with altered synaptic plasticity, altered neuronal morphology, and altered synaptic structure and function. Mechanistic assessment demonstrates that widespread proteomic changes and altered dynamics of the secretory pathway underly the synaptic defects. The phenotype of ATase1 and ATase2 overexpressing mice is reminiscent of SLC25A1, SLC13A5 and AT-1 overexpressing models. Therefore, when taken together, our results support the argument that the intracellular citrate/acetyl-CoA pathway, with the ATases acting as the last output, is immediately connected to the pathogenesis of certain rare forms of autism spectrum disorder.

## Introduction

Neurons heavily depend on the ability of the secretory pathway to deliver correctly folded polypeptides to the periphery of the cell for the assembly, maintenance, and normal functioning of synapses. Quality control as well as protein sorting mechanisms within the endoplasmic reticulum (ER) are in place to ensure that only correctly folded polypeptides can successfully engage and transition through the secretory pathway. Studies in model systems indicate that ER acetylation is required for positive selection of correctly folded polypeptides in the ER lumen as well as induction of ER-specific autophagy (reticulophagy) to dispose of protein aggregates that form within the organelle [1, 2]. ER acetylation requires the coordinated activity of three essential proteins: AT-1/SLC33A1, ATase1/NAT8B and ATase2/NAT8. AT-1 is a membrane transporter that ensures transfer of cytosolic acetyl-CoA into the ER lumen while ATase1 and ATase2 are ER-resident Nε-lysine acetyltransferases that transfer the acetyl group from the acetyl-CoA donor to the amino-end of the acceptor lysine [1, 2].

Defective ER acetylation, as caused by mutations and gene duplication events, is associated with severe human disease phenotypes spanning from developmental delay with premature death, peripheral forms of neuropathy, autism-spectrum disorder (ASD) with intellectual disability and progeria-like dysmorphism [2]. Importantly, duplications involving 3q25.31 (harboring *AT-1/SLC33A1/AT-1*) and 2p13.1 (harboring *ATase1/NAT8B* and *ATase2/NAT8*) are specifically associated with ASD and intellectual disability with dysmorphism (National Organization for Rare Disorders database at https://rarediseases.org/; see also [3–11]). The above disease associations can be modeled in the mouse. Indeed, neuron-specific overexpression of AT-1 causes an ASD-like phenotype, while systemic overexpression causes a segmental progeria-like phenotype [12–14]. Finally, mutant mice with homozygous or heterozygous inactivation of AT-1 display developmental arrest and spastic paraplegia, respectively [15]. In essence, balanced ER-based Nε-lysine acetylation appears to be vitally important for neuron biology.

The antiporter activity of AT-1 allows ER acetylation to respond to changes in citrate/acetyl-CoA availability in the cytosol. Key to this crosstalk is the citrate transport activity of the mitochondria membrane transporter, SLC25A1, and the plasma membrane transporter, SLC13A5 [2]. Importantly, gene duplications events affecting 22q11.21 (harboring *SLC25A1*) and 17p13.1 (harboring *SLC13A5*) are also associated with ASD and intellectual disability with dysmorphism (National Organization for Rare Disorders database at https://rarediseases.org/; see also [16–19]). As with AT-1, neuron-specific overexpression of SLC25A1 or SLC13A5 in the mouse causes an ASD-like phenotype [20, 21]. Biochemical analysis has clearly positioned the ER acetylation machinery down-stream of the SLC25A1/SLC13A5 network supporting the conclusion that the intracellular flux of citrate and acetyl-CoA is particularly important for neuron biology [20–22].

In this study, we report the generation and phenotypic characterization of mice with neuron-specific overexpression of ATase1 or ATase2. The animals display autistic-like behaviors with altered synaptic plasticity and altered neuronal morphology. We also show that both models exhibit altered synaptic structure and function with widespread proteomic changes. Finally, we demonstrate that changes in dynamics of the secretory pathway underly the synaptic defects.

## Materials and methods

### Transgenic mouse generation

Camk2a-tTA;TRE-ATase1 (referred to as ATase1 nTg thereafter) and Camk2a-tTA;TRE-ATase2 (referred to as ATase2 nTg thereafter) mice were generated as previously described [12, 20, 21]. Human cDNA was isolated from ATase1- and ATase2-pCMV6 plasmids (Origene; RC215647 for ATase1/NAT8B and RC202157 for ATase2/NAT8) using PCR and subsequently subcloned into pTRE-Tight plasmid (Takara Bio, Inc.). The resulting pTRE-Tight-ATase1 and pTRE-Tight-ATase 2 plasmids were linearized with XhoI and then injected into C57BL/6 J mice (The Jackson Laboratory; Stock No. 000664). These mice were subsequently crossed with B6.Cg-Tg(Camk2atTA)1Mmay/DboJ (Camk2a-tTA) mice (The Jackson Laboratory; Stock No. 007004) to create ATase1 and ATase2 neuron transgenic (nTg) mice. Genotyping from tail DNA was conducted using these specified primers: ATase1 forward (5′-GCTCGTTTAGTGAACCGTCAGAT-3’), ATase1 reverse (5′-CTCCTGGTATTTGCGGATGTGAT-3’); ATase2 forward (5′-GCTCGTTTAGTGAACCGTCAGAT-3’), ATase2 reverse (5′-CTCCTGGTATTTGCGGATGTGA-3’); Camk2a-tTA forward (5′-CGCTGTGGGGCATTTTACTTTAG-3′), and Camk2a-tTA reverse (5′-CATGTCCAGATCGAAATCGTC-3’).

### Animals

The University Laboratory Animal Resources provided standardized cages for mice housing. Each cage had one to five littermates and was supplied with regular chow and water *ad libitum*. All the experiments were done according to the National Institutes of Health Guide for the Care and Use of Laboratory Animals and approved by the UW-Madison committee for animal care (protocol #M005120). The TRE-ATase1, TRE-ATase2, and Camk2a-tTA transgenes were all bred as heterozygous. Throughout the investigation, wild type (WT), non-transgenic littermates were employed as controls. Animals were used from multiple litters at random. Both males and females were studied. The age and sex of animals at time of experimentation are specified in the figure legends. Investigators were not blind to the genotype of the animals. However, behavioral assessment, mass spectrometry analysis, Imaris-assisted morphology analysis, and computer-assisted multi-electrode array analysis were conducted in a blinded fashion.

### Behavior testing

The behavioral assays were all performed at the Behavioral Testing Service of the Waisman Center (University of Wisconsin-Madison, Madison, WI, USA). Prior to each behavior assay, mice were placed in the testing room for a 30-min acclimation period. The performed behavioral assays consisted of the marble burying assay, social interaction, novel object recognition, and fear conditioning paradigm, all of which have been previously described [12, 20, 21]. Additionally, the following tests were conducted:

#### Open field exploration

The mice were individually removed from their cages and placed in the center of an arena for a single 30-min open field session. The Omnitech Fusion system used photobeams to monitor and record the animal’s placement during the session. Measured variables consisted of the total distance covered (in centimeters), the number of vertical activity episodes, the total time spent in ambulation (in seconds), and the distance traveled within the centroid (in centimeters). Data were recorded using the Omnitech Fusion system with a center ratio zone map.

#### Light/dark exploration

Mice were placed in a divided arena for a period of 10 min. Each mouse was allocated one session, with the time spent in the arena (in seconds) and the number of entrances into the light and darkened areas of the arena being recorded.

#### Jumping, grooming, and digging

For jumping, video recordings of the mice in their cages were taken; a red light was used to visualize the dark cycle. The number of jumps performed by each mouse was quantified within a 20-min time frame. For spontaneous grooming and digging, the mice were placed in a new empty cage with standard bedding. Grooming and digging behaviors were quantified within a 20-min time frame using Stopwatch+ [23, 24].

### Primary neuron cultures

Primary neurons were obtained from P0 pups using a papain dissociation kit (Worthington Biochemical Corporation; LK003150) and cultured for a maximum of 28 days. The micro-dissected cortex and hippocampus were stained. To analyze synapse formation, Syn-1 (MACS; 130-119-358; 1:200), Psd-95 (Thermo Fisher; MAI-045; 1:500) primary antibodies and phalloidin stain (Abcam; ab176753) were used for immunofluorescence analysis. Images were collected using a Nikon A1 inverted confocal microscope with NIS-Elements AR version 5.11.01 software using 405 nm blue channel, 488 nm green channel, 561 nm red channel, and 640 nm far red laser wavelengths. The imaging process involved capturing multi-z-stack images with dimensions of 1024 × 1024 pixels and 15 z-steps every 0.2 µm. This was achieved using a 60x oil objective with a numerical aperture of 1.4 and a pixel size of 0.21 µm. The pinhole size used was 72.8 µm. Imaris (Version 9.5) was used to import the .nd2 images and convert them to the .ims format. The Filament Tracer module with the Autopath method was used to trace dendrites from the soma, with the thinnest diameter being 1.5 μm, and dendrite seed points within 30 µm of the soma were removed. Dendrite spines were then identified, with the seed point diameter being 0.7 μm and the maximum length being 7 µm. To measure dendritic branching, a Sholl analysis was conducted with 1 µm spaced spheres. To assess synapse formation, spots with a diameter of 2 µm were fitted to the pre-and post-synaptic markers; co-localized spots were analyzed using Imaris software.

### Microelectrode array activity

For multi-electrode array (MEA), 48-well MEA plates (Axion Biosystems; M768-tMEA-48B-5) were pre-coated with PDL (Thermo Fisher; A3890401) for 1 h, rinsed three times with sterile water, and then dried overnight. On the following day, postnatal hippocampal and cortical cell suspensions were prepared with mouse laminin (Thermo Fisher; 23017015; 1 µg/mL) supplementation. 50,000 cells per well in a volume of 5 to 10 µL were placed in the middle of the well and incubated for one hour at 37 °C and 5% CO_2_; after 1 h plating, 200 µL of neuron culture media were added to each well. Cells were maintained at the same temperature and CO_2_ level with a half media change every 3-4 days. Spontaneous activity recordings were performed every 7 days in vitro (DIV) with the use of Axion Biosystems Maestro pro multifunctional system. Neural Real Time configured for continuous spontaneous activity data were collected for a period of 10 min using Axion Navigator software. A band-pass filter (3,000 Hz to 200 Hz) was applied utilizing variable threshold spikes detection. This was employed at ±6 standard deviation of the root mean squared of the background noise. An active electrode was determined based on a minimum spike rate of 5 spikes per minute, while a mature network was defined as having at least 8 out of 16 active electrodes. The inter-spike interval threshold was utilized to detect bursts in mature networks, with a maximum inter-spike interval of 100 ms and a minimum of 5 spikes. Additionally, network bursts were detected in mature networks with a minimum number of 50 spikes and a minimum of 35% participating electrodes, while also applying a synchronicity window of 20 ms. The neural excitability was represented by mean firing rate (Hz) and burst rate (Hz), whereas network synchronization was represented by network burst rate (Hz) and network synchronicity (index value between 0 and 1).

### Electrophysiology

Extracellular recordings of field Excitatory Postsynaptic Potentials (fEPSPs), long-term potentiation (LTP), and long-term depression (LTD) were collected as per previously described methods [20, 21] with specific modifications. The slice preparation solution consisted of N-methyl-D-glucamine (NMDG, 93 mM), NaH_2_PO_4_ (1.2 mM), KCl (2.5 mM), NaHCO_3_ (30 mM), glucose (25 mM), sodium ascorbate (5 mM), sodium pyruvate (3 mM), thiourea (2 mM), HEPES (20 mM), CaCl_2_ (0.5 mM), and MgSO_4_ (10 mM). Also, the following composition was used for artificial cerebrospinal fluid (aCSF) recording: NaCl (124 mM), NaH_2_PO_4_ (1.25 mM), KCl (3 mM), NaHCO_3_ (26 mM), glucose (15 mM), sodium ascorbate (0.8 mM), CaCl_2_ (2.5 mM) and MgSO_4_ (1.3 mM). When saturated with carbogen, all the solutions were buffered to a pH of 7.3 and the osmolality was confirmed to be between 294 and 297 mOsm. Coronal slices were subject to recordings using fire polished borosilicate glass recording pipettes, which were filled with aCSF (3–5 MΩ) and a Pt/Ir concentric bipolar stimulating electrode. The potentiation and depression of the recordings were determined by dividing the average fEPSP slope over the last 10 min of the recording by the average 10 min of baseline immediately prior to induction of either LTP or LTD.

### Western blotting

Western blotting was performed as previously described [12, 20, 21]. The primary antibodies used in the study were as follows: NAT8/NAT8B (Abcepta; AD4957c) and β-actin (CST; 3700; 1:1,000). The following secondary antibodies were used for infrared imaging on a LICOR Odyssey Infrared Imaging System (LI-COR Biosciences): Goat anti-mouse (926-32210) and donkey anti-rabbit (926-68070). Supplementary Figure 6 in the manuscript shows the original uncropped images from Western blot experiments.

### Histology and immunostaining

Histology, immunostaining techniques, and Golgi staining were conducted according to the instructions described earlier [12, 15, 20, 21, 25, 26]. Klüver-Barrera staining was conducted on slices embedded in 10 µm of paraffin using the EMS kit (Electron Microscopy Sciences; 26681). Primary antibodies used for immunostaining included Iba1 (Abcam; ab178846), Gfap (Abcam;ab4674), Myelin Basic Protein (Abcam; ab40390; 1:200), MOG (Abcam; ab233549), Olig2 (Millipore; AB9610; 1:300), NAT8/NAT8b (Abcepta; AD4957c), and NeuN (Millipore; ABN91MI; 1:1,000).

The upright Leica DM4000 B microscope, equipped with a 10x air objective and 100x oil objective, was used to acquire bright-field images, and Image-Pro version 6.3 was utilized for image processing. All slides with fluorescent labeling were imaged on a Nikon A1 inverted confocal microscope, utilizing NIS-Elements AR version 5.11.01 software and Galvano scan head technology, with laser wavelengths of blue channel (405 nm), green channel (488 nm), red channel (561 nm), and far red (640 nm). For the slides stained with NAT8/NAT8B/NeuN, single z-slice images were obtained at a resolution of 1024 × 1024 pixels using a 10x air objective (NA = 0.3; 1.24 µm/pixel) and 60x oil objective (NA = 1.4; 0.21 µm/pixel) with pinhole size of 197.95 µm. For MBP/NeuN-stained slides, single z-slice images (1024 × 1024 pixels; 1.24 µm/pixel) were acquired usage of 10x air objective (NA = 0.3) at a pinhole size of 72.8 µm. The 10x objective (NA = 0.3) and pinhole size of 72.8 µm for MBP/MOG/Olig2/NeuN-stained slides produced single z-slice images with (1024 × 1024 pixels; 1.24 µm/pixel) resolution.

Microglial Iba1-positive cells were quantified using ImageJ (Version 1.52). This was achieved by creating binary images through an intensity threshold and then counting the objects with a Particle Analyzer tool. For Golgi staining images, pre-processing was done in ImageJ (Version 1.52) by inverting and then subtracting background three consecutive times with pixel sizes of 100, 50, and 25. The resulting images were stored in .tiff files, imported into Imaris (Bitplane; Version 9.5) and converted into the native .ims format. To analyze the secondary dendritic branches, the Autopath method of the Filament Tracer module was used. Branches were semi-manually traced with 0.25 µm and automatic dendrite volume detection was allowed. A mask was created to remove signals from reconstructed dendrites, allowing for the detection of branched spines with a minimum dendrite diameter of 0.25 μm and a maximum dendrite length of 5 μm. For further analysis, the dendrite spine density (measured in spines per 10 µm of dendrite length) and spine volume (measured in µm^3^) data were used.

### ManAz (sialylation) assay

Primary cortical neuron cells were cultured onto coverslips in a 24-well plate at a density of 10,000 cells per well and allowed to grow for 2 weeks in a neurobasal A medium. Following this, Click-iT ManNAz was introduced at a concentration of 50 μM and the cells were incubated at 37 °C for 48 h. After the incubation period, the cells were carefully washed in PBS, fixed with 4% PFA and subsequently blocked using BSA solution. Cells were stained using a combination of Click-iT reaction buffer, cell buffer additive and alkyne dye for 30 min. Coverslips were mounted on microscope slides and images captured in a Nikon A1. The presence of ManAz at the cell membrane was measured through synapse volume changes with the aid of Imaris software and through quantifying fluorescent intensity using ImageJ.

### Proteomics

#### Protein extraction and digestion

Cortical and hippocampal tissues were resuspended in 8 M urea lysis buffer (50 mM Tris-HCl, PH = 8), containing protease and phosphatase inhibitors (Thermo Fisher). Protein concentration was determined using a commercial bicinchoninic acid (BCA) protein assay. Proteins from all samples were reduced with 5 mM dithiothreitol (DTT) for 30 min at 37 °C and then alkylated with 15 mM iodoacetamide (IAA) for 45 min at room temperature (RT) in the dark. The samples were quenched by 5 mM DTT for 10 min at RT. The protein mixture was diluted with 50 mM Tris-HCl (pH=8) to a final urea concentration of ≤ 1 M before the addition of trypsin (Promega). Complete tryptic digestion was achieved using a trypsin-to-protein ratio of 1:50 (w/w) at 37 °C overnight. The digestion was quenched by acidification with 10% trifluoroacetic acid to a final pH below 3. Digested samples were desalted using Sep-Pak C18 cartridges (Waters) and eluted first with 0.1% formic acid (FA) in 50% acetonitrile (ACN) and then with 0.1% FA in 80% ACN. A Pierce Quantitative Colorimetric Peptide Assay was used to determine peptide concentration, with 200 μg peptides aliquoted for each sample and used for DiLeu isobaric labeling.

#### DiLeu labeling

Two sets of 12-plex *N, N*-dimethyl leucine (DiLeu) tags were used to label 24 samples, which included 12 cortex tissue samples and 12 hippocampus tissue samples. The DiLeu activation solution was prepared with anhydrous *N*,*N*-dimethylformamide, 4-(4,6-dimethoxy-1,3,5-triazin-2-yl)-4-methylmorpholinium tetrafluoroborate and *N*-methylmorpholine at 0.6x molar ratio to DiLeu tags. The tags were activated by vortexing the mixture of tags and activation solution for 45 min at RT. The supernatant of activation solution was then mixed with peptide samples reconstituted in 0.5 M triethylammonium bicarbonate buffer, followed by vortexing at RT for 2 h. The reaction was quenched with 5% hydroxylamine to a final concentration of 0.25%, and the mixtures were vortexed at RT for 10 min. Labeled peptide samples were combined at a ratio of 1:1:1:1:1:1:1:1:1:1:1:1 and dried *in vacuo*. Pooled samples underwent cleaning with strong cation exchange (SCX) according to the manufacturer’s protocols. To enhance proteome coverage and improve detection of low-abundance proteins, off-line high pH (HpH) fractionation was performed prior to liquid chromatography-tandem mass spectrometry (LC-MS/MS) analysis. Each DiLeu labeled set obtained 8 combined fractions after HpH fractionation.

#### LC-MS/MS

The samples were analyzed using a Q Exactive HF orbitrap mass spectrometer (Thermo Fisher Scientific) coupled with a Dionex UltiMate 3000 UPLC system. Each sample was dissolved in 0.1% FA before undergoing chromatographic separation on a 15 cm C18 column (1.7 μm, 130 Å, Waters). Mobile phase A consisted of water with 0.1% FA, while mobile phase B was composed of ACN with 0.1% FA. Separation was achieved with gradient elution of 4% to 40% mobile phase B over 120 min at a flow rate of 0.3 µL/min. Full MS data were acquired within a mass scan range of *m/z* 300–1500 at a resolution of 60 K, with an automatic gain control (AGC) target of 1 ×106 and a maximum injection time (IT) of 100 ms. For data-dependent acquisition tandem mass spectrometry (DDA-MS2), the top 20 precursor ions were selected for MS2 fragmentation at a resolution of 60 K, AGC target of 1 ×105, maximum IT of 200 ms, isolation width of 1.0 Da, fixed first mass at *m/z* 110, normalized collision energy (NCE) of 30, and dynamic exclusion was 45 seconds.

#### Data analysis

Protein identification and quantification were performed using MaxQuant (version 1.5.2.8). The collected mass spectral data were searched against the UniProt mouse reviewed database (November 2023). Trypsin was set as the digesting protease with two missed cleavages allowed. DiLeu labeling on peptide N termini and lysine residues (+145.12801 Da), and carbamidomethylation of cysteine residues (+57.02146 Da) were selected as static modifications. Oxidation of methionine residues (+15.99492 Da), and deamidation of asparagine and glutamine residues (+0.98402 Da) were defined as variable modifications. The first search peptide tolerance and main search peptide tolerance were set at 20 ppm and 4.5 ppm, respectively. Match between runs was enabled, and other parameters were set as default. Reporter ion intensities were normalized following correction of isotopic impurities, and subsequent analysis was performed by Perseus (1.6.15.0) and DAVID bioinformatics resources.

### Statistical analysis

The data were analyzed using GraphPad Prism version 9.5.0, and all results are reported as mean ± standard deviation unless otherwise noted. For comparisons between two groups, an unpaired t-test was utilized. Ordinary one-way or two-way ANOVA was used for comparisons between three or more groups, with subsequent multiple comparison tests of either Tukey-Kramer (for all group comparisons) or Dunnett’s (for comparisons to a single control group). If ANOVA testing did not reveal any differences in sex, the data were merged to improve visual representation. The specifics of each statistical test are outlined in the figure legends. Grubb’s test was employed to eliminate outlier values identified at a significance level of *P* < 0.05. Statistical significance was determined as *P* < 0.05. All experiments were conducted at least two independent times with successful replication.

## Results

### ATase1 nTg and ATase2 nTg mice display autistic-like behavior

**Fig. 1:**
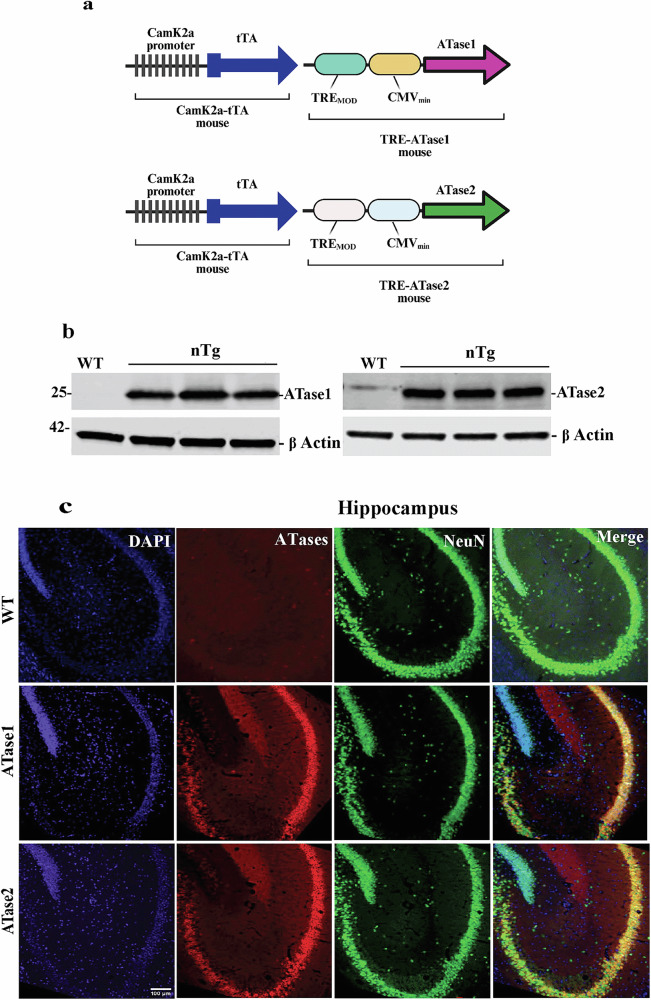
ATase nTg mice overexpress the ATases in neurons. **a** ATase nTg mice were generated using an inducible, neuron-specific overexpression Tet-Off system operated by the CamK2a promoter. **b** Protein expression of human ATase1 and ATase2 in the brain. Mice were 3-month-old males. **c** Paraffin-embedded immunostaining. Mice were 3-month-old males.

To investigate the role of the ER-based acetyltransferases, ATase1 and ATase2, in the development of ASD, we generated C57BL/6 J mice overexpressing human ATase1 or ATase2 in neurons. Specifically, we used a previously characterized Tet-Off system driven by the Camk2 promoter (Fig. 1a) [12, 20, 21]. As expected, the animals (here referred to as ATase1 nTg and ATase2 nTg) displayed a robust expression of the ATase protein in the brain as detected by an antibody directed against human ATases (Fig. 1b). Importantly, the expression was specifically observed within neurons as assessed by co-localization with the neuronal marker NeuN (Fig. 1c).

**Fig. 2:**
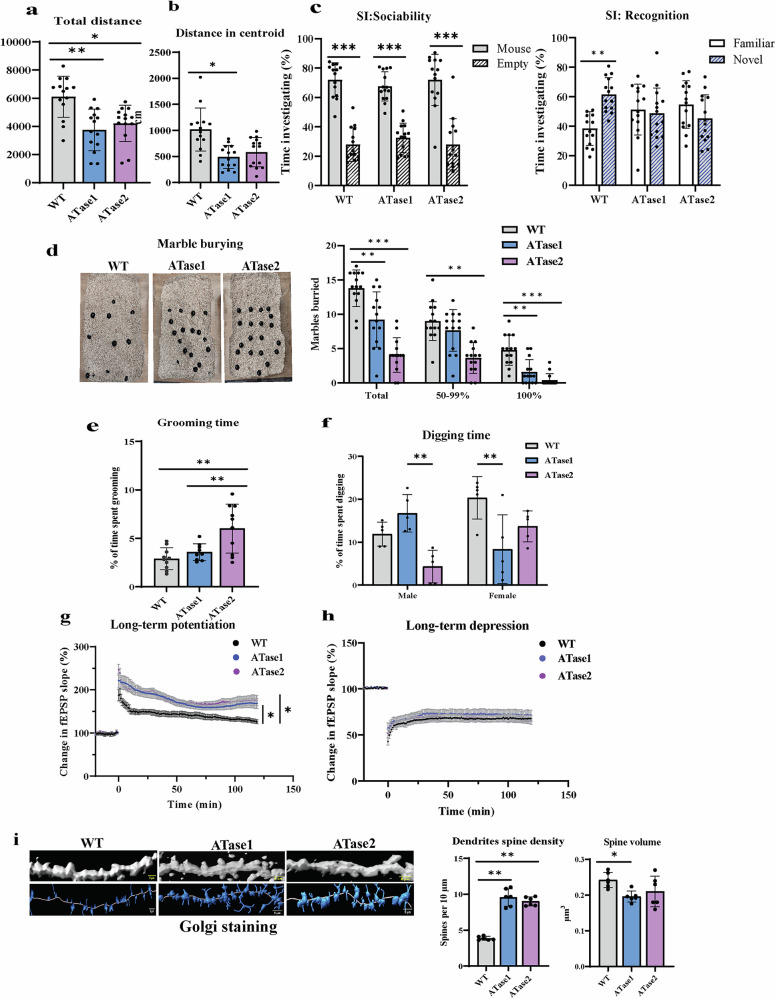
ATases nTg mice display autistic-like behaviors and altered synaptic plasticity. **a,** **b** Open field test showing (a) total distance and (b) distance in centroid area. **c** Social interaction (SI) paradigms using the sociability (*left*) and recognition (*right*) tests. **d** Marble burying assay. **e** Grooming time paradigm. **f** Digging time paradigm. Data are represented as mean ± SD. *P ≤ 0.05, **P ≤ 0.005 and ***P ≤ 0.0005 via two-way ANOVA (Genotype x Sex). WT, n = 14; nTg, n = 14; equal number of males and females. All mice were 3–4 months old. **g** Theta-burst (3x) LTP in hippocampal brain. **h** LTD in hippocampal brain. Data are represented as mean ± SEM. *P ≤ 0.05 via Student’s t-test of the average potentiation value in the last 10 min; n = 10 slices per genotype (3 males and 3 females from WT, ATase1 and ATase2 nTg mice, respectively). Mice were 3–4 months old. (**i**) Golgi staining of CA1 pyramidal neuron apical dendrites. Representative images along with unbiased dendrite and spine reconstructions are displayed. Dendrite spine density and reconstructed spine’s volume data are shown as mean ± SD with each value indicating one mouse. *P ≤ 0.05, **P ≤ 0.005 via Student’s t-test; n = 6 mice per genotype (3 males and 3 females) at 3 months of age.

To determine whether the neuron-specific overexpression of either acetyltransferase would impact the behavior of the animals, we conducted a series of formalized behavioral studies targeting different hippocampal- and cortical- as well as social-based behaviors, similar to those already assessed in the SLC25A1 nTg, SLC13A5 nTg, and AT-1 nTg models [12, 20, 21]. In the open field paradigm, both nTg mice spent significantly less time exploring the arena overall (Fig. 2a) and displayed a clear aversion of the central area (Fig. 2b). Within the social interaction paradigms, both the WT and the nTg mice displayed preferential interest in the mouse rather than the empty cup (Fig. 2c; *left panel*); however, in contrast to WT mice, the nTg did not discriminate between the familiar and the novel mouse (Fig. 2c; *right panel*). Additionally, both ATase1 and the ATase 2 nTg mice exhibited reduced marble burying behavior (Fig. 2d). Small differences among the two nTg models were also observed, with ATase2 nTg mice spending more time with self-grooming (Fig. 2e) and ATase1 nTg displaying divergent digging behavior (Fig. 2f). Finally, about 50% of ATase1 nTg and 28% of ATase2 nTg mice displayed repetitive jumping behavior (Video 1). Neither ATase1 nTg nor ATase2 nTg mice displayed noteworthy differences from WT controls when assessed for vertical activity or ambulatory time within the open field paradigm, or overall exploration within the light-dark paradigm (Supplementary Figure 1a–c). Furthermore, no differences were observed with the novel object recognition and fear conditioning paradigms (Supplementary Figure 1d, e).

Overall, our ATase1 and ATase2 nTg mice displayed several behavioral abnormalities that have been observed in other rodent models of ASD, including SLC25A1 nTg, SLC13A5 nTg and AT-1 nTg [12, 20, 21].

### ATase1 nTg and ATase2 nTg mice have altered synaptic structure and function

To begin investigating the mechanistic basis of the phenotype, we conducted a comprehensive histological assessment of the brain but, as expected, we did not observe gross differences (Supplementary Figure 2–4). Next, we performed hippocampal brain slice electrophysiology to determine synaptic plasticity of the Schaffer collateral pathway. Indeed, it is well documented that a balance between long-term potentiation (LTP) and long-term depression (LTD) must be present to ensure normal behavioral functions [27, 28]. Using a theta burst stimulation paradigm to induce LTP, we observed increased potentiation in both ATase nTg mice as compared to WT controls (Fig. 2g). In contrast to LTP, no significant LTD alterations were observed (Fig. 2h).

In light of the above behavioral and LTP abnormalities, we performed several experiments to examine neuron morphology and function. To begin, we used the Golgi stain to visualize neurons in vivo. Although no gross alterations were observed (Supplementary Figure 5), high-magnification imaging of CA1 pyramidal neuron apical dendrites revealed an increase in dendrite spine density in both ATase1 nTg and ATase2 nTg mice (Fig. 2i). These in vivo alterations are reminiscent of the SLC25A1 nTg, SLC13A5 nTg and AT-1 nTg models [12, 20, 21], and are consistent with the abnormal synaptic plasticity demonstrated by enhanced LTP in the Schaffer collateral to CA1 synapse.

**Fig. 3:**
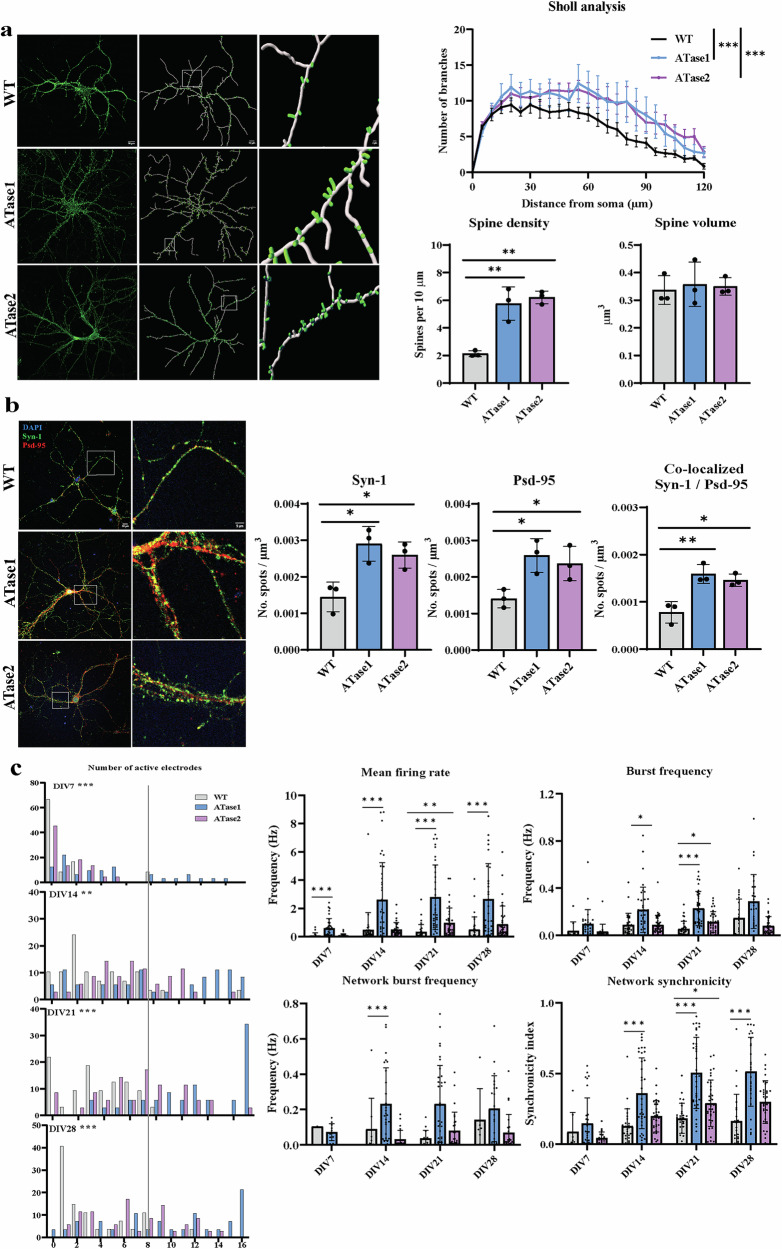
Primary cortical neurons from ATases nTg mice exhibit increased dendritic branching, spine density, and spontaneous activity. **a** Morphological analysis of cultured neurons at 15 days in vitro. Images show phalloidin staining (*left*) and computer-assisted reconstruction (*right*), accompanied by quantification from a sample size of 3 mice per genotype. Sholl analysis data are reported as the mean ± SEM. ***P ≤ 0.0005 with two-way ANOVA. Spine density and volume data are presented as mean ± SD with each data point representing one mouse. **P ≤ 0.005 with Student’s t-test. **b** Immunostaining of cultured neurons for pre/postsynaptic markers, Syn-1 and Psd-95, at 15 days in vitro. Puncta were measured with a diameter of 2 µm and co-localization was determined if the spots were within 1 µm of each other. The results are presented as mean ± SD, with each data point representing one mouse. A total of 3 WT, 3 ATase1 and 3 ATase2 nTg mice were included in the study. *P ≤ 0.01 and **P ≤ 0.005 via Student’s t-test. **c** Spontaneous neuronal activity using MEA. Histogram results showing the number of active electrodes per network represented as relative frequency in per cent (*left*). A vertical line was used to demarcate eight active electrodes, which is considered the minimum value for a mature network. **P ≤ 0.005 and ***P ≤ 0.0005 using the Mann-Whitney comparing frequency distributions. Spontaneous activity was also measured by mean firing rate, burst frequency, network burst frequency, and synchronicity index (*right*). Each individual data-point represents a distinct network of cultured neurons and displays activity in at least 8 out of 16 electrodes. *P ≤ 0.05, **P ≤ 0.005, and ***P ≤ 0.0005 using mixed effects analysis with Sidak’s multiple comparison test. The data were collected from six mice per genotype.

Next, we cultured primary neurons to study neuron morphology as well as synapse formation and function. At 15 days in vitro (DIV), cortical neurons of both ATase1 nTg and ATase2 nTg exhibited increased dendritic branching compared to WT littermates as demonstrated by the Sholl analysis (Fig. 3a). These morphological alterations were accompanied by a marked increase in dendritic spine density with overall normal spine volume, similar to what was found using the Golgi stain (Fig. 3a). To determine spontaneous synapse formation between adjacent neurons in vitro, we performed immunostaining for pre- and post-synaptic markers and assessed for their co-localization. We observed an increase in puncta formation of the pre-synaptic marker Syn-1 and the post-synaptic marker Psd-95 in both ATase1 nTg and ATase2 nTg cortical neurons, as compared to WT littermates (Fig. 3b). Importantly, we also observed increased co-localization of Syn-1/Psd-95 puncta (Fig. 3b). Taken together, the above results support the conclusion that cortical neurons from our transgenic mice have a higher density of synapses.

To assess the function of these synapses, we plated our cortical neurons on multielectrode arrays (MEAs) and monitored their spontaneous electrical activity every 7 days up to 28 DIV. The number of active electrodes, which reflect spontaneous activity of the MEA network, gradually increased over time in all genotypes. Interestingly, ATase1 nTg and ATase2 nTg neurons had a higher number of active electrodes, suggesting that the network matured more rapidly compared to WT littermates (Fig. 3c). Additionally, the transgenic neurons exhibited higher firing rate, higher burst frequency, and higher network synchronicity at different time points (Fig. 3c). When comparing the two nTg models, ATase1 overexpressing neurons appeared more active than ATase2 overexpressing neurons across all MEA-functions (Fig. 3c).

**Fig. 4:**
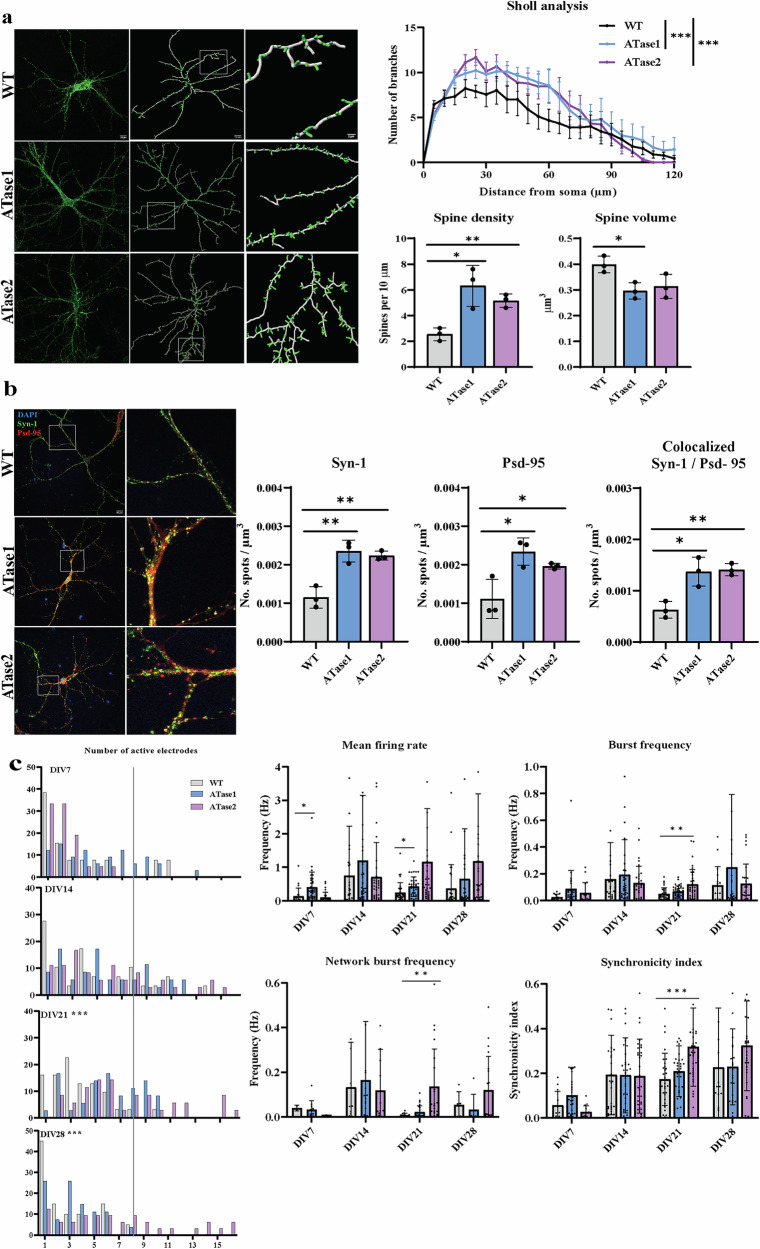
Primary hippocampal neurons from ATases nTg mice exhibit increased dendritic branching, spine density, and spontaneous activity. **a** Morphological analysis of cultured neurons at 15 days in vitro. Images show phalloidin staining (*left*) and computer-assisted reconstruction (*right*), accompanied by quantification from a sample size of 3 pups per genotype. Sholl analysis data are reported as the mean ± SEM. ***P ≤ 0.0005 through two-way ANOVA. Spine density and spine volume data are presented as mean ± SD with each data point representing one mouse. *P ≤ 0.05; **P ≤ 0.005 with Student’s t-test. **b** Immunostaining of cultured neurons for pre/postsynaptic markers, Syn-1 and Psd-95, at 15 days in vitro. Puncta were measured with a diameter of 2 µm and co-localization was determined if the spots were within 1 µm of each other. The results are presented as mean ± SD, with each data point representing one mouse. A total of 3 WT, 3 ATase1 and 3 ATase2 nTg mice were included in the study. *P ≤ 0.05; **P ≤ 0.005 using a Student’s t-test. **c** Spontaneous activity using MEA. Histogram results showing the number of active electrodes per network represented as relative frequency in per cent (*left*). A vertical line was used to demarcate eight active electrodes, which is considered the minimum value for a mature network. ***P ≤ 0.0005 using the Mann-Whitney test comparing frequency distributions. Spontaneous activity was also measured by mean firing rate, burst frequency, network burst frequency and synchronicity index (*right*). Each individual data-point represents a distinct network of cultured neurons and displays activity in at least 8 out of 16 electrodes. *P ≤ 0.05; **P ≤ 0.005; ***P ≤ 0.0005 using mixed effects analysis with Sidak’s multiple comparison test. The data were collected from six mice per genotype.

To confirm the above results with cortical neurons, we plated hippocampal neurons and repeated the same studies. Again, we observed an increased number of dendritic branches together with a higher number of dendritic spine density (Fig. 4a). These results were also accompanied by increased Syn-1 and Psd-95 puncta as well as Syn-1/Psd-95 co-localized puncta (Fig. 4b). ATase1 nTg and ATase2 nTg hippocampal neurons also had a higher percentage of active electrodes on the MEA plates at DIV21 and DIV28 (Fig. 4c). However, we did not observe the robust change in activity that we observed with cortical neurons (Fig. 4c).

Overall, these data demonstrate that ATase1 nTg and ATase2 nTg primary neurons in vitro exhibit robust alterations in both morphology and activity, as reflected by increased dendritic branching and dendritic spine density with a concomitant increase in neural excitability. Importantly, the in vitro data match very closely the in vivo data with increased spine density and increased LTP.

### ATase1 and ATase2 nTg mice display differential changes in the hippocampal and cortical proteome highlighting an increased efficiency of the secretory pathway

**Fig. 5:**
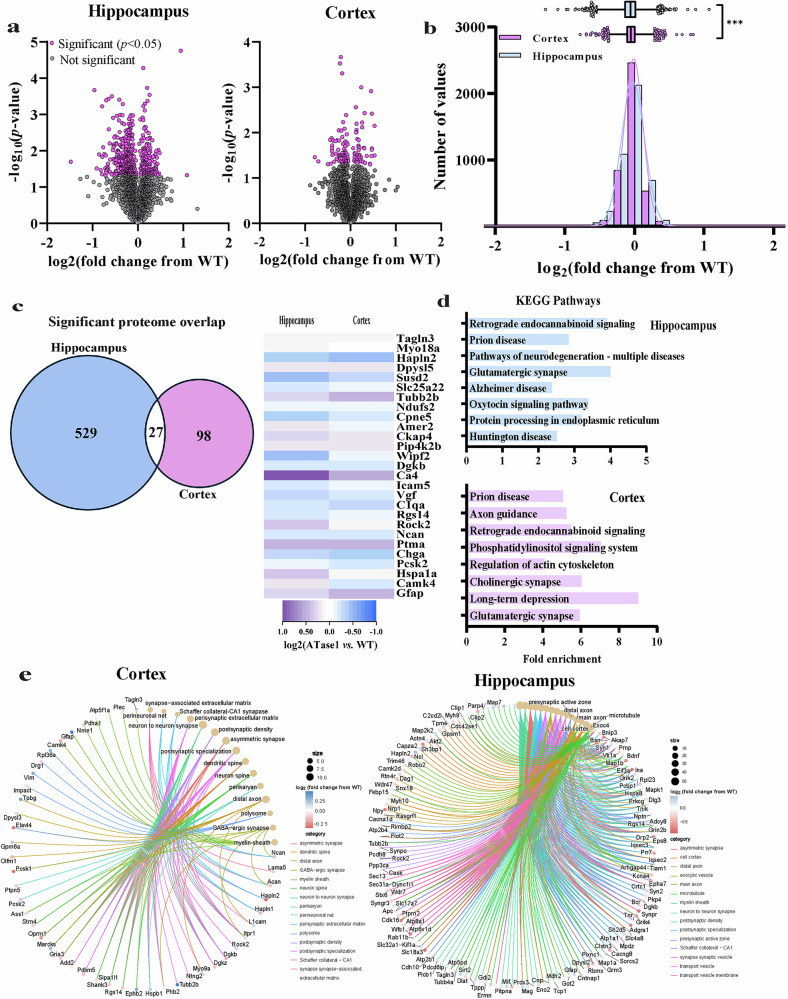
The hippocampus and cortex of ATase1 nTg mice exhibit global proteomic alterations. **a** The volcano plots depict all quantified proteins in the hippocampus and cortex of ATase1 nTg mice compared to WT controls. Proteins that showed statistically significant changes are highlighted in magenta (556 in the hippocampus and 125 in the cortex; *P* < 0.05 via Two-sample tests); all other proteins are represented in grey. The data were collected from a sample of 4 male mice per genotype at 4 months of age. **b** Histogram and overlaid Gaussian distribution displaying the distribution of log2 fold changes from WT in hippocampus and cortex for all proteins. The accompanying box and whisker plot depicts the 25^th^ and 75^th^ percentiles as the boxed area, the mean as the middle line, and the 1^st^/99^th^ percentiles as the whiskers. ***P ≤ 0.0005 using the Kolmogorov–Smirnov test. **c** Significantly changed proteins that overlap between the hippocampus and the cortex. All proteins represented in magenta in panel **a** are included. The heatmap displays the expression profile of the 27 proteins that are common. **d** Fold enrichment of KEGG pathways determined from significantly changed proteins compared to WT. The top 8 categories with the highest enrichment scores, filtered by a FDR score of *P* < 0.01, are presented. **e** Gene networks of proteins significantly altered in the ATase1 nTg hippocampus (*left*) and cortex (*right*). Plots were generated through an overrepresentation analysis using the GO cellular component function database. The dot size within the network is scaled to the number of overlapping proteins within that category. The top 15 results, filtered with a FDR score of 0.05, are displayed.

To dissect the molecular mechanism of the ATase1 and ATase2 nTg phenotypes, we performed quantitative proteomics of the brain and detected 4318 and 4030 proteins in the hippocampus and cortex, respectively. In the case of ATase1 nTg mice, 556 and 125 proteins in the hippocampus and cortex, respectively, had a statistically significant change in expression compared to WT controls (*P* < 0.05) (Fig. 5a). The hippocampal response appeared to be more dramatic and largely different from the cortex. First, the overall distribution was different, with more proteins altered in the hippocampus than the cortex (Fig. 5a, b). Second, the number of proteins shared across datasets was quite small (27 out of 556 for the hippocampus and 125 for the cortex; Fig. 5c). However, they were all associated with developmental processes that included cell migration- and adhesion-related events. Finally, KEGG pathway analysis of all the significantly changed proteins revealed distinct categories between the hippocampus and cortex (Fig. 5d). To understand the possible cellular implication of the proteomic changes, we constructed gene-network plots with the significantly changed proteins using the gene ontology (GO) cellular component function database (Fig. 5e). To limit our analysis, only the top 15 most stringent functions were considered, including neuron to neuron synapse, neuron and dendritic spine, postsynaptic density and specialization, and Schaffer collateral to CA1 synapse organization. Therefore, despite differential proteomic changes between the hippocampus and cortex, the cellular functions affected were quite similar and closely related to the mouse phenotype. In other words, although hippocampus and cortex responded differently to the overexpression of ATase1, the affected cellular functions were remarkably similar (Fig. 5e).

**Fig. 6:**
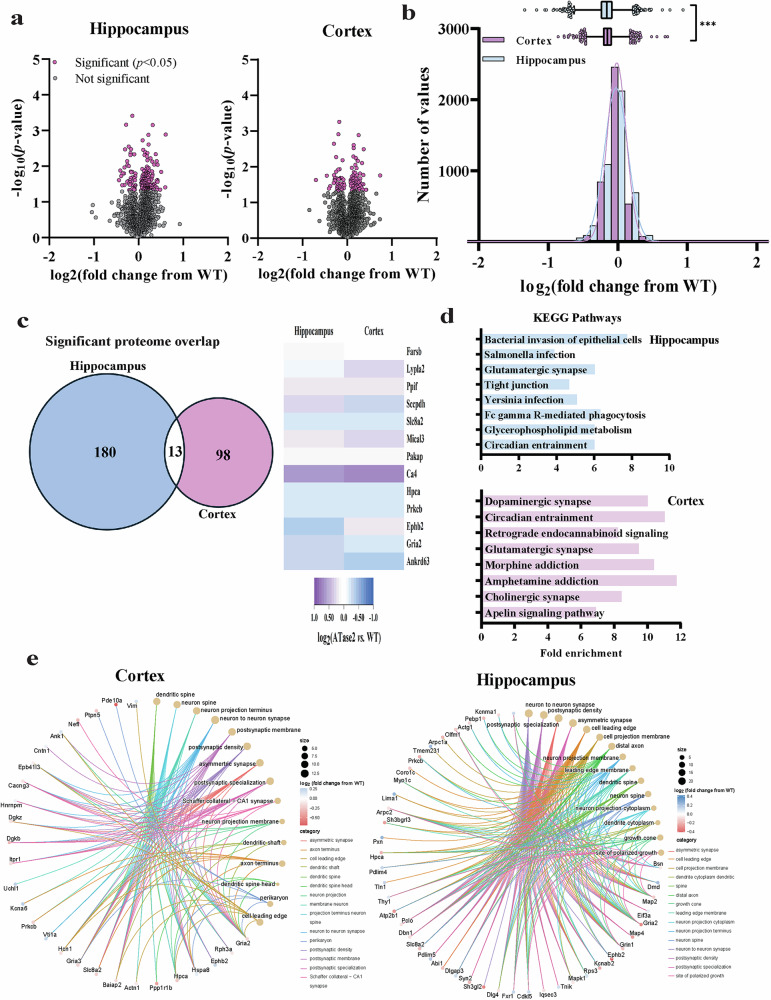
The hippocampus and cortex of ATase2 nTg mice exhibit global proteomic alterations. **a** The volcano plots depict the quantified proteins in the hippocampus and cortex of ATase2 nTg mice compared to WT controls. Proteins that showed statistically significant changes are highlighted in magenta (193 in the hippocampus and 111 in the cortex; *P* < 0.05 by Two-samples tests); all other proteins are represented in grey. The data were collected from a sample of 4 male mice per genotype at 4 months of age. **b** Histogram and overlaid Gaussian distribution displaying the distribution of log2 fold changes from WT in hippocampus and cortex for all proteins. The accompanying box and whisker plot depicts the 25^th^ and 75^th^ percentiles as the boxed area, the mean as the middle line, and the 1^st^/99^th^ percentiles as the whiskers. ***P ≤ 0.0005 using the Kolmogorov–Smirnov test. **c** Significantly changed proteins that overlap between the hippocampus and the cortex. All proteins represented in magenta in panel **a** are included. The heatmap displays the expression profile of the 13 proteins that are common. **d** Fold enrichment of KEGG pathways determined from significantly changed proteins compared to WT. The top 8 categories with the highest enrichment scores, filtered by a FDR score of *P* < 0.01, are presented. **e** Gene networks of proteins significantly altered in the ATase2 nTg hippocampus (*left*) and cortex (*right*). Plots were generated through an overrepresentation analysis using the GO cellular component function database. The dot size within the network is scaled to the number of overlapping proteins within that category. The top 15 results, filtered with a FDR score of 0.05, are displayed.

In the case of ATase2 nTg mice, 193 and 111 proteins in the hippocampus and cortex, respectively, had a statistically significant change in expression compared to WT controls (*P* < 0.05) (Fig. 6a). Although the number of significantly altered proteins between hippocampus and cortex were closer than in the ATase1 nTg model (see Fig. 5c), we still observed differences among the two tissues. Indeed, we only found 13 significantly altered proteins being shared across tissues (Fig. 6c), and the KEGG pathway analysis of the significantly changed proteins revealed distinct categories (Fig. 6d). However, as with ATase1 nTg mice, the gene-network plots identified very similar cellular component functions being enriched (Fig. 6e). They included several processes involved with the assembly and activity of synapses and were closely related to the mouse phenotype.

**Fig. 7:**
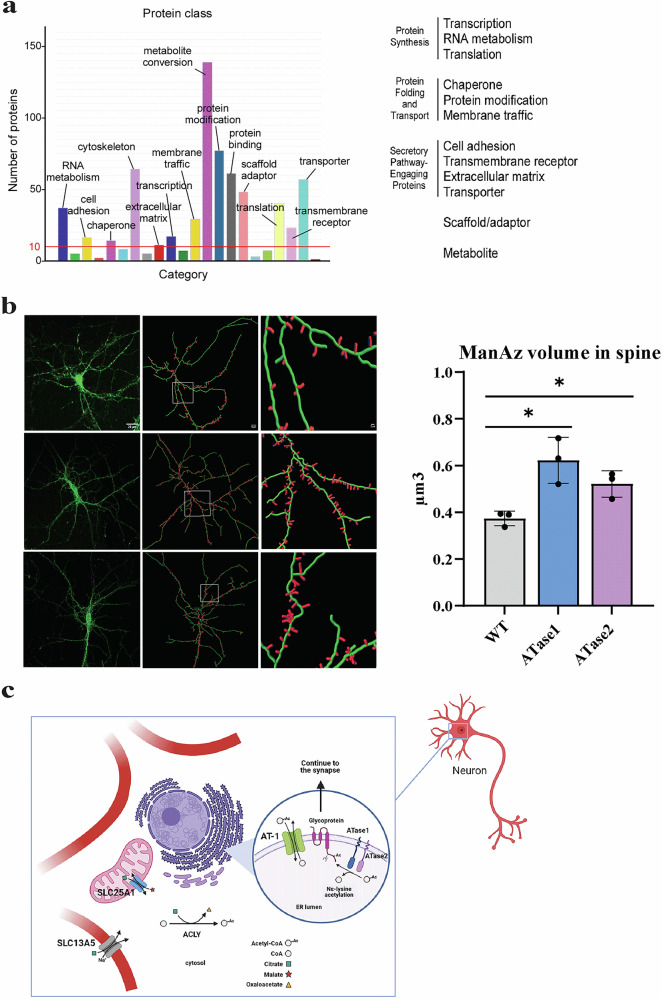
ATase1 nTg and ATase2 nTg display altered engagement of the secretory pathway. **a** PANTHER Functional classification of all significantly changed proteins compared to WT. Only proteins with unambiguous classification are shown. **b** Visualization of ManAz incorporation on cell-surface glycoproteins by spine volume quantification. Data are mean ± SD with each data point representing one mouse. *P ≤ 0.05 from WT littermates through Student’s t-test. **c** Model integrating the different nTg mice that we generated. Image was assembled using BioRender.

Overall, the unbiased proteomic analysis revealed differences across tissues (hippocampus *vs* cortex) and models (ATase1 nTg *vs* ATase2 nTg); however, it also demonstrated a common response of the brain to the overexpression of either ATase that impacted basic neuronal events involved in development, outgrowth of dendritic branches and formation of functional synapses. Importantly, all these biological events heavily depend on the engagement and efficiency of the secretory pathway and are closely related to the biochemical and biological functions of the ATases [2]. Indeed, collectively, most changed proteins across models could be functionally classified into three major biochemical groups: protein synthesis, protein folding and transport, and secretory pathway-engaging proteins (Fig. 7a).

To experimentally test the apparent involvement of the secretory pathway delineated by the unbiased proteomic analysis, we labeled isolated primary neurons in culture with ManAz, an azido-modified precursor of sialic acid that is used to label N-linked glycosylation of cell surface proteins [12, 29, 30]. ManAz labeling of cortical neurons demonstrated a marked accumulation of ManAz glycoproteins on the dendritic spines of both ATase1 and ATase2 nTg mice (Fig. 7b). It is worth remembering that the initial oligosaccharide structure that is added by the oligosaccharyltransferase within the ER lumen undergoes major modifications as glycoproteins move through the Golgi apparatus and receive Golgi-specific sugars, such as sialic acid, which is added only in the *trans*-Golgi and *trans*-Golgi network [31]. Therefore, the increased incorporation of ManAz onto cell surface glycoproteins reflects an increased transition of nascent glycoproteins through the secretory pathway of ATase-overexpressing neurons.

Overall, both the proteomic and the ManAz data (Figs. 5–7) highlight a clear involvement of the secretory pathway with increased translocation of nascent glycoproteins to the neuronal surface, thus supporting the morphological changes observed (Figs. 3–4).

## Discussion

In this study, we report that mice with overexpression of human ATase1 and ATase2 in forebrain neurons display autistic-like behaviors with altered synaptic plasticity and altered neuronal morphology. We also show that both models present altered synaptic structure and function with widespread proteomic changes. Finally, we demonstrate that changes in dynamics of the secretory pathway underly the synaptic defects. There is no consensus on the exact behavioral and electrophysiological aberrations that define ASD in rodents. Considerable overlap also exists with other neurodevelopmental diseases such as Attention Deficit/Hyperactivity Disorder (ADHD) [32–34]. However, the phenotype of ATase1 and ATase2 overexpressing mice is reminiscent of other ASD-like models including SLC25A1, SLC13A5 and AT-1 nTg mice [12, 20, 21, 33–36]. Therefore, although we recognize the limitations of modeling human behavioral hallmarks in the mouse, we view the ATase1 and ATase2 nTg phenotypes as autistic-like.

ER acetylation requires AT-1, ATase1 and ATase2 [1, 2]. AT-1 is the ER membrane transporter that supplies acetyl-CoA to the lumen of the ER while ATase1 and ATase2 are the two ER-luminal acetyltransferases that utilize acetyl-CoA for Nε-lysine acetylation [2]. Functional biochemistry revealed that the ATases interact with the oligosaccharyltransferase complex within the ER membrane to acetylate correctly folded glycoproteins while studies in model systems revealed that ER-acetylation is required for positive selection of correctly folded polypeptides in the ER lumen [1, 2]. In essence, the ER acetylation machinery functions as a novel branch of the more general ER quality control/sorting machinery [1, 2]. Neuron-specific overexpression of AT-1 in the mouse caused increased flux of nascent glycoproteins through the secretory pathway, increased dendritic branch and spine formation, and altered synaptic plasticity [12]. Neuron-specific overexpression of ATase1 or ATase2 caused a similar outcome (present study). In conclusion, the phenotype of the AT-1 nTg, ATase1 nTg and ATase2 nTg mice is consistent with the biochemical and biological functions attributed to the ER acetylation machinery (Fig. 7c).

Proteomic assessment of the ATase nTg models highlights a clear involvement of proteins related to dynamics of the secretory pathway, from transcription and translation to folding and quality control, transport across the secretory pathway, and assembly of relevant cell-surface protein complexes. The ultimate effect is demonstrated by the increased incorporation of ManAz into nascent glycoproteins, increased formation of dendritic branches and spines, and altered synaptic plasticity. Highly represented within our datasets were cell-surface membrane-bound glycoproteins that are fundamental to establishing cellular connections such as Hapln1, Hapln2, Can, Lama5, Ncan, Dag1, and Pcdh8. Also represented were proteins that are relevant for synaptic activity such as Gria3, Cacna1d, Slc17a7, Slc32a1, Grik2, and Slc8a2, as well as scaffolding proteins such as Pdlim5 and Shank3, which are also important for synaptic activity. Importantly, unbiased glycoproteomics of mice with increased, AT-1 sTg, or reduced, AT-1^S113R/+^, ER acetylation revealed drastic changes in the sialylation and fucosylation of transiting glycoproteins in the brain, thus supporting our interpretation of the present data [37].

The ER acetylation acts down-stream of the citrate/acetyl-CoA flux to ensure intracellular metabolic crosstalk and functional adaptation of the secretory pathway to the cellular metabolic state [2]. Consistently, mice with neuron-specific overexpression of the two citrate transporters, SLC25A1 and SLC13A5, developed biochemical, cellular, and behavioral features that are overall similar to AT-1, ATase1, and ATase2 nTgs [20, 21]. In other words, a consistent outcome emerges from all the mice with increased ER acetylation that we have so far generated, thus suggesting that the intracellular citrate/acetyl-CoA pathway, with the ATases acting as the last output, is immediately connected to the pathogenesis of certain rare forms of ASD (Fig. 7c).

Although they share enzymatic properties, ATase1 and ATase2 are differentially regulated at the transcriptional and translational level [38]. This partially divergent regulation of the ATases also emerged when we analyzed the phenotype of Atase1^-/-^ and Atase2^-/-^ mice where we observed different adaptive changes within the acetyl-proteome and acetyl-CoA metabolism [39]. The proteomic analysis of the brain of ATase1 nTg and ATase2 nTg again revealed a divergent response with different sets of proteins being affected in the two models. Nevertheless, the phenotype of the two models was strikingly similar. Specifically, the phenotype of Atase1^-/-^ mice was similar to Atase2^-/-^ mice [39], and the phenotype of ATase1 nTg mice was similar to ATase2 nTg mice (present study). In other words, although they might have evolved to target different substrates, the two acetyltransferases work in concert to regulate similar cellular functions. This is not entirely surprising given their genetic origin, believed to be from a gene duplication event, and their sequence similarity. As with our prior models, AT-1 nTg, SLC25A1 nTg, and SLC13A5 nTg [12, 20, 21], the proteomic assessment revealed distinct changes in proteins between the cortex and the hippocampus. Nevertheless, very similar pathways were affected as revealed by the GO cellular component and KEGG pathway analyses. Furthermore, primary neurons derived from cortex or hippocampus exhibited similar morphological changes as also reflected by similar alterations at the neuronal excitability and synaptic plasticity level. Therefore, while different adaptive responses are at play, they are working in concert to regulate similar cellular functions.

In conclusion, we have demonstrated that overexpression of either ATase1 or ATase2 in mouse forebrain neurons results in an autistic-like phenotype with altered synaptic plasticity and altered neuronal morphology. Mechanistically, the phenotype was linked to aberrant engagement of the secretory pathway.

## Supplementary information

Supplementary Movie 1

Supplementary Information

## Data Availability

The proteomic data supporting this study have been deposited to the PRIDE under ID number PXD051786. All remaining data and supplementary information relevant to this study are included in this paper.

## References

[1] Farrugia MA, Puglielli L. Nepsilon-lysine acetylation in the endoplasmic reticulum - a novel cellular mechanism that regulates proteostasis and autophagy. J Cell Sci. 2018;131:jcs221747. 30446507 10.1242/jcs.221747 PMC6262770

[2] Fernandez-Fuente G, Rigby MJ, Puglielli L. Intracellular Citrate/acetyl-CoA flux and endoplasmic reticulum acetylation: connectivity is the answer. Mol Metab. 2023;67:101653. 36513219 10.1016/j.molmet.2022.101653 PMC9792894

[3] Krumm N, O’Roak BJ, Karakoc E, Mohajeri K, Nelson B, Vives L, et al. Transmission disequilibrium of small CNVs in simplex autism. Am J Hum Genet. 2013;93:595–606. 24035194 10.1016/j.ajhg.2013.07.024 PMC3791263

[4] Krumm N, Turner TN, Baker C, Vives L, Mohajeri K, Witherspoon K, et al. Excess of rare, inherited truncating mutations in autism. Nat Genet. 2015;47:582–8. 25961944 10.1038/ng.3303 PMC4449286

[5] Poultney CS, Goldberg AP, Drapeau E, Kou Y, Harony-Nicolas H, Kajiwara Y, et al. Identification of small exonic CNV from whole-exome sequence data and application to autism spectrum disorder. Am J Hum Genet. 2013;93:607–19. 24094742 10.1016/j.ajhg.2013.09.001 PMC3791269

[6] Rizzu P, Haddad BR, Vallcorba I, Alonso A, Ferro MT, Garcia-Sagredo JM, et al. Delineation of a duplication map of chromosome 3q: a new case confirms the exclusion of 3q25-q26.2 from the duplication 3q syndrome critical region. Am J Med Genet. 1997;68:428–32. 9021016

[7] Ounap K, Ilus T, Bartsch O. A girl with inverted triplication of chromosome 3q25.3 –> q29 and multiple congenital anomalies consistent with 3q duplication syndrome. Am J Med Genet A. 2005;134:434–8. 15793836 10.1002/ajmg.a.30134

[8] Francke U. Clinical syndromes associated with partial duplications of chromosomes 2 and 3: dup(2p),dup(2q),dup(3p),dup(3q). Birth Defects Orig Artic Ser. 1978;14:191–217. 365267

[9] Fineman RM, Buyse M, Morgan M. Variable phenotype associated with duplication of different regions of 2p. Am J Med Genet. 1983;15:451–6. 6881212 10.1002/ajmg.1320150310

[10] Fryns JP, Kleczkowska A, Kenis H, Decock P, Van den Berghe H. Partial duplication of the short arm of chromosome 2 (dup(2)(p13—p21) associated with mental retardation and an Aarskog-like phenotype. Ann Genet. 1989;32:174–6. 2573314

[11] Sawyer JR, Jones E, Hawks FF, Quirk JG Jr., Cunniff C. Duplication and deletion of chromosome band 2(p21p22) resulting from a familial interstitial insertion (2;11)(p21;p15). Am J Med Genet. 1994;49:422–7. 8160737 10.1002/ajmg.1320490414

[12] Hullinger R, Li M, Wang J, Peng Y, Dowell JA, Bomba-Warczak E, et al. Increased expression of AT-1/SLC33A1 causes an autistic-like phenotype in mice by affecting dendritic branching and spine formation. J Exp Med. 2016;213:1267–84. 27242167 10.1084/jem.20151776 PMC4925020

[13] Peng Y, Shapiro SL, Banduseela VC, Dieterich IA, Hewitt KJ, Bresnick EH, et al. Increased transport of acetyl-CoA into the endoplasmic reticulum causes a progeria-like phenotype. Aging Cell. 2018;17:e12820. 30051577 10.1111/acel.12820 PMC6156544

[14] Murie M, Peng Y, Rigby MJ, Dieterich IA, Farrugia MA, Endresen A, et al. ATase inhibition rescues age-associated proteotoxicity of the secretory pathway. Commun Biol. 2022;5:173. 35217767 10.1038/s42003-022-03118-0 PMC8881600

[15] Peng Y, Li M, Clarkson BD, Pehar M, Lao PJ, Hillmer AT, et al. Deficient import of Acetyl-CoA into the ER lumen causes neurodegeneration and propensity to infections, inflammation, and cancer. J Neurosci. 2014;34:6772–89. 24828632 10.1523/JNEUROSCI.0077-14.2014 PMC4019794

[16] Mooneyham KA, Holden KR, Cathey S, Dwivedi A, Dupont BR, Lyons MJ. Neurodevelopmental delays and macrocephaly in 17p13.1 microduplication syndrome. Am J Med Genet A. 2014;164A:2887–91. 25123844 10.1002/ajmg.a.36708

[17] Carvalho CM, Vasanth S, Shinawi M, Russell C, Ramocki MB, Brown CW, et al. Dosage changes of a segment at 17p13.1 lead to intellectual disability and microcephaly as a result of complex genetic interaction of multiple genes. Am J Hum Genet. 2014;95:565–78. 25439725 10.1016/j.ajhg.2014.10.006 PMC4225592

[18] Kylat RI. 22q11.2 microduplication: an enigmatic genetic disorder. J Pediatr Genet. 2018;7:138–42. 30105124 10.1055/s-0038-1655754 PMC6087476

[19] Wenger TL, Miller JS, DePolo LM, de Marchena AB, Clements CC, Emanuel BS, et al. 22q11.2 duplication syndrome: elevated rate of autism spectrum disorder and need for medical screening. Mol Autism. 2016;7:27. 27158440 10.1186/s13229-016-0090-z PMC4859984

[20] Rigby MJ, Orefice NS, Lawton AJ, Ma M, Shapiro SL, Yi SY, et al. Increased expression of SLC25A1/CIC causes an autistic-like phenotype with altered neuron morphology. Brain. 2022;145:500–16. 35203088 10.1093/brain/awab295 PMC9014753

[21] Rigby MJ, Orefice NS, Lawton AJ, Ma M, Shapiro SL, Yi SY, et al. SLC13A5/sodium-citrate co-transporter overexpression causes disrupted white matter integrity and an autistic-like phenotype. Brain Commun. 2022;4:fcac002. 35146426 10.1093/braincomms/fcac002 PMC8823335

[22] Fernandez-Fuente G, Overmyer KA, Lawton AJ, Kasza I, Shapiro SL, Gallego-Munoz P, et al. The citrate transporters SLC13A5 and SLC25A1 elicit different metabolic responses and phenotypes in the mouse. Commun Biol. 2023;6:926. 37689798 10.1038/s42003-023-05311-1 PMC10492862

[23] Kalueff AV, Stewart AM, Song C, Berridge KC, Graybiel AM, Fentress JC. Neurobiology of rodent self-grooming and its value for translational neuroscience. Nat Rev Neurosci. 2016;17:45–59. 26675822 10.1038/nrn.2015.8 PMC4840777

[24] Pond HL, Heller AT, Gural BM, McKissick OP, Wilkinson MK, Manzini MC. Digging behavior discrimination test to probe burrowing and exploratory digging in male and female mice. J Neurosci Res. 2021;99:2046–58. 34048600 10.1002/jnr.24857 PMC9066774

[25] Pehar M, O’Riordan KJ, Burns-Cusato M, Andrzejewski ME, del Alcazar CG, Burger C, et al. Altered longevity-assurance activity of p53:p44 in the mouse causes memory loss, neurodegeneration and premature death. Aging Cell. 2010;9:174–90. 20409077 10.1111/j.1474-9726.2010.00547.x PMC2848983

[26] Peng Y, Kim MJ, Hullinger R, O’Riordan KJ, Burger C, Pehar M, et al. Improved proteostasis in the secretory pathway rescues Alzheimer’s disease in the mouse. Brain. 2016;139:937–52. 26787453 10.1093/brain/awv385 PMC4805081

[27] Gebicke-Haerter PJ. The computational power of the human brain. Front Cell Neurosci. 2023;17:1220030. 37608987 10.3389/fncel.2023.1220030 PMC10441807

[28] Stacho M, Manahan-Vaughan D. The intriguing contribution of hippocampal long-term depression to spatial learning and long-term memory. Front Behav Neurosci. 2022;16:806356. 35548697 10.3389/fnbeh.2022.806356 PMC9084281

[29] Jacobs CL, Yarema KJ, Mahal LK, Nauman DA, Charters NW, Bertozzi CR. Metabolic labeling of glycoproteins with chemical tags through unnatural sialic acid biosynthesis. Methods Enzymol. 2000;327:260–75. 11044989 10.1016/s0076-6879(00)27282-0

[30] Baskin JM, Prescher JA, Laughlin ST, Agard NJ, Chang PV, Miller IA, et al. Copper-free click chemistry for dynamic in vivo imaging. Proc Natl Acad Sci USA. 2007;104:16793–7. 17942682 10.1073/pnas.0707090104 PMC2040404

[31] Hirschberg CB, Robbins PW, Abeijon C. Transporters of nucleotide sugars, ATP, and nucleotide sulfate in the endoplasmic reticulum and Golgi apparatus. Annu Rev Biochem. 1998;67:49–69. 9759482 10.1146/annurev.biochem.67.1.49

[32] Leo D, Gainetdinov RR. Transgenic mouse models for ADHD. Cell Tissue Res. 2013;354:259–71. 23681253 10.1007/s00441-013-1639-1 PMC3785710

[33] Lewis MH, Tanimura Y, Lee LW, Bodfish JW. Animal models of restricted repetitive behavior in autism. Behav Brain Res. 2007;176:66–74. 16997392 10.1016/j.bbr.2006.08.023 PMC3709864

[34] Silverman JL, Yang M, Lord C, Crawley JN. Behavioural phenotyping assays for mouse models of autism. Nat Rev Neurosci. 2010;11:490–502. 20559336 10.1038/nrn2851 PMC3087436

[35] Berg JM, Lee C, Chen L, Galvan L, Cepeda C, Chen JY, et al. JAKMIP1, a novel regulator of neuronal translation, modulates synaptic function and autistic-like behaviors in mouse. Neuron. 2015;88:1173–91. 26627310 10.1016/j.neuron.2015.10.031 PMC4829343

[36] Ryan BC, Young NB, Crawley JN, Bodfish JW, Moy SS. Social deficits, stereotypy and early emergence of repetitive behavior in the C58/J inbred mouse strain. Behav Brain Res. 2010;208:178–88. 19941908 10.1016/j.bbr.2009.11.031 PMC2822076

[37] Dieterich IA, Cui Y, Braun MM, Lawton AJ, Robinson NH, Peotter JL, et al. Acetyl-CoA flux from the cytosol to the ER regulates engagement and quality of the secretory pathway. Sci Rep. 2021;11:2013. 33479349 10.1038/s41598-021-81447-6 PMC7820588

[38] Rigby MJ, Ding Y, Farrugia MA, Feig M, Cortese GP, Mitchell H, et al. The endoplasmic reticulum acetyltransferases ATase1/NAT8B and ATase2/NAT8 are differentially regulated to adjust engagement of the secretory pathway. J Neurochem. 2020;154:404–23. 31945187 10.1111/jnc.14958 PMC7363514

[39] Rigby MJ, Lawton AJ, Kaur G, Banduseela VC, Kamm WE, Lakkaraju A, et al. Endoplasmic reticulum acetyltransferases Atase1 and Atase2 differentially regulate reticulophagy, macroautophagy and cellular acetyl-CoA metabolism. Commun Biol. 2021;4:454. 33846551 10.1038/s42003-021-01992-8 PMC8041774

