# Supplementary material for: Increased expression of ATase1/NAT8B or ATase2/NAT8 in the mouse results in an autistic-like phenotype with altered dendritic branching and spine formation
Source: Mol Psychiatry. 2025 Sep 24;31(1):1–15. doi: 10.1038/s41380-025-03228-1 (PMC12700795; doi:10.1038/s41380-025-03228-1)
Supplement: Supplementary file 2 — Supplementary Information [file 41380_2025_3228_MOESM2_ESM.pdf]

SUPPLEMENTARY INFORMATION

Increased expression of ATase1/NAT8B or ATase2/NAT8 in the mouse results in an autistic-like phenotype with altered dendritic branching and spine formation

Balagangadharan Kalimuthu[1,2], Haiyan Lu[3], Angelique Steenhagen[1,2], Qiping Dong[2,4], Mitchell Gray[5], Michael J. Rigby[1,2,8], Andreas Endresen[1,2,9], Qiang Chang[2,4], Lingjun Li[3,5], and Luigi Puglielli[1,2,6,7*]

[1]Department of Medicine, University of Wisconsin-Madison, Madison, WI, USA

[2]Waisman Center, University of Wisconsin-Madison, Madison, WI, USA

[3]School of Pharmacy, University of Wisconsin-Madison, Madison, WI, USA

[4]Department of Medical Genetics and Neurology, University of Wisconsin-Madison, Madison, WI, USA

[5]Department of Chemistry, University of Wisconsin-Madison, Madison, WI, USA

[6]Geriatric Research Education Clinical Center, Veterans Affairs Medical Center, Madison, WI, USA

[7]Department of Neuroscience, University of Wisconsin-Madison, Madison, WI, USA

[8]Present address: Department of Neurology, Mayo Clinic, Rochester, MN, USA

[9]Present address: Boehringer-Ingelheim, Ridgefield, CT, USA

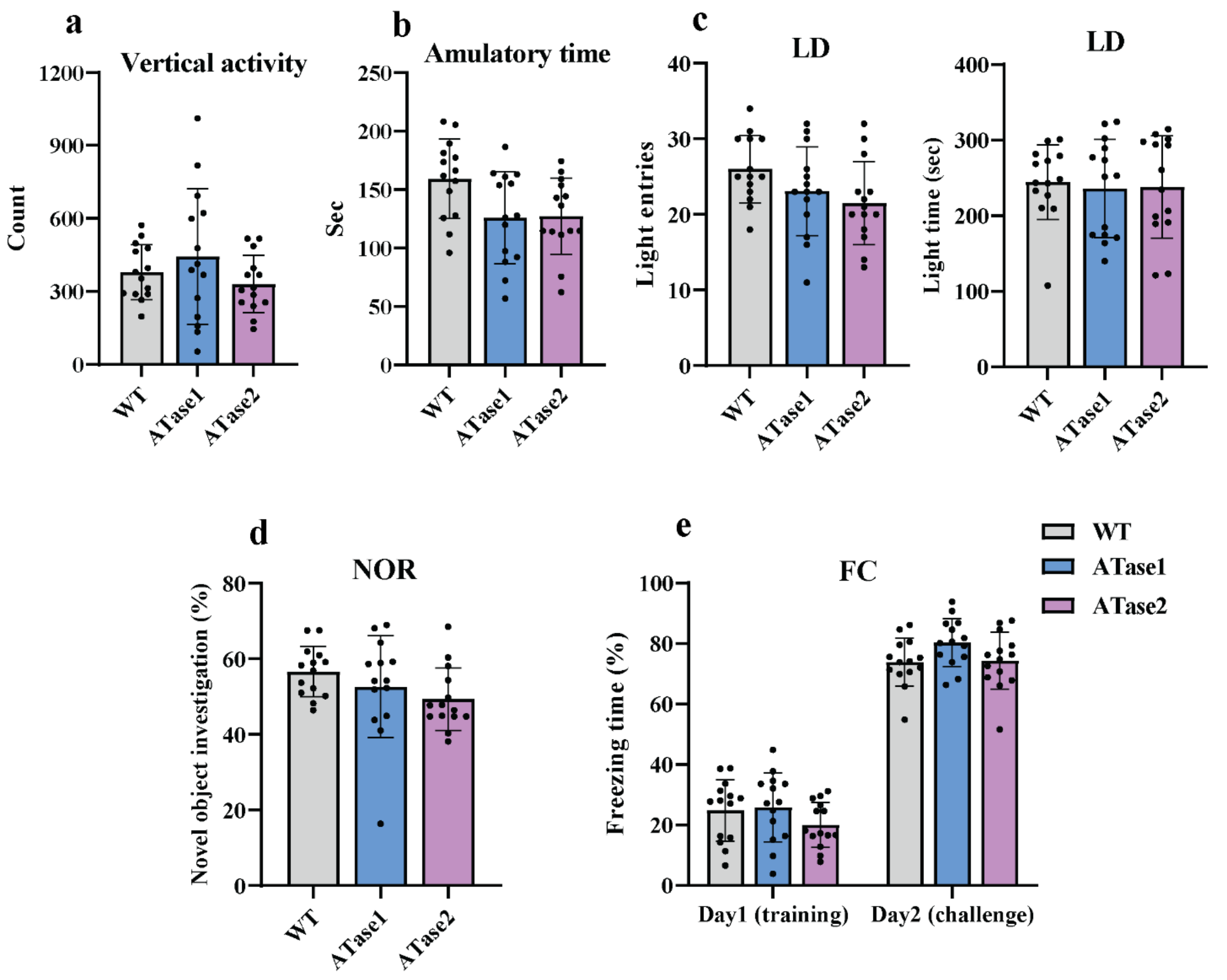

Supplementary Figure 1. Behavioral assessment in ATase1 nTg and ATase2 nTg mice. (**a**) Vertical activity. (**b**) Ambulatory time. (**c**) Light-Dark exploration. (**d**) Novel object recognition. (**e**) Fear conditioning. Data are mean ± SD. WT, n = 14; nTg, n = 14; equal number of males and females. All mice were 3–4 months old.

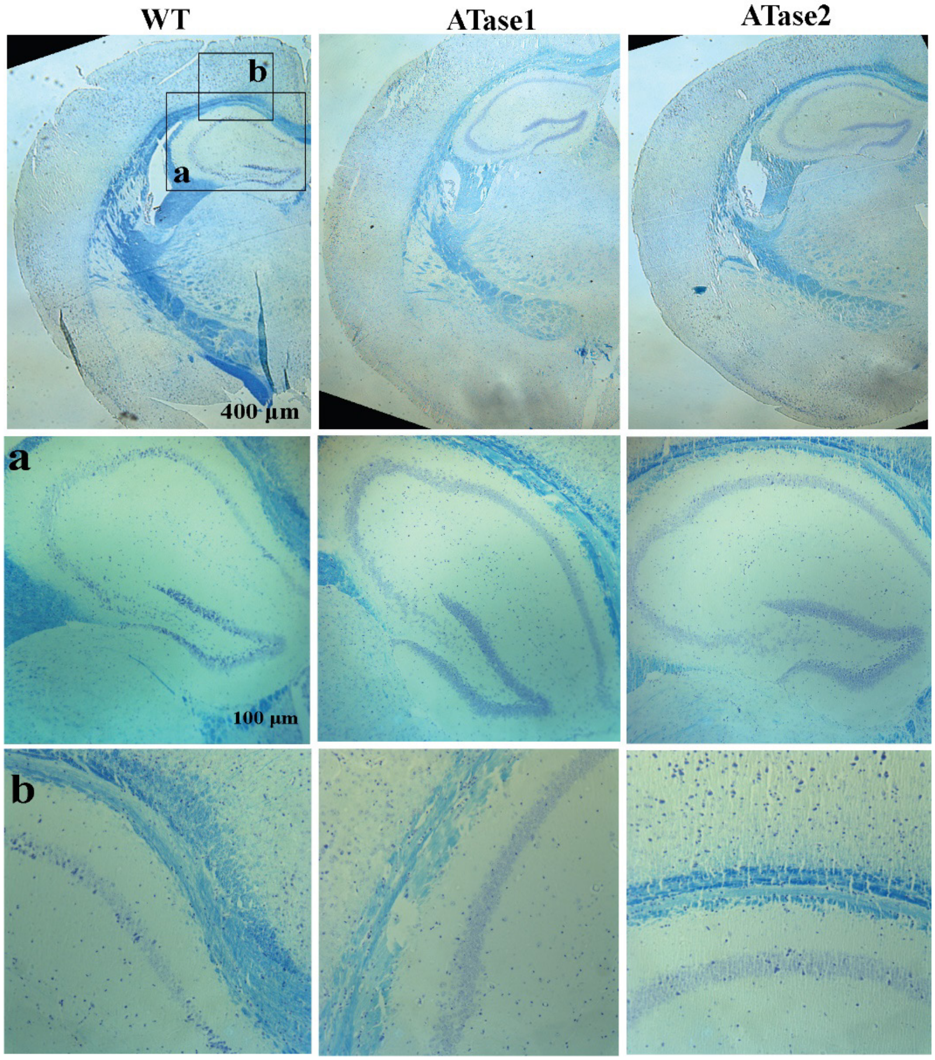

Supplementary Figure 2. ATase1 nTg and ATase2 nTg mice do not demonstrate histologically visible alterations of white matter integrity. Kluver-Barerra staining of paraffin-embedded brain. All mice were 7-month-old females.

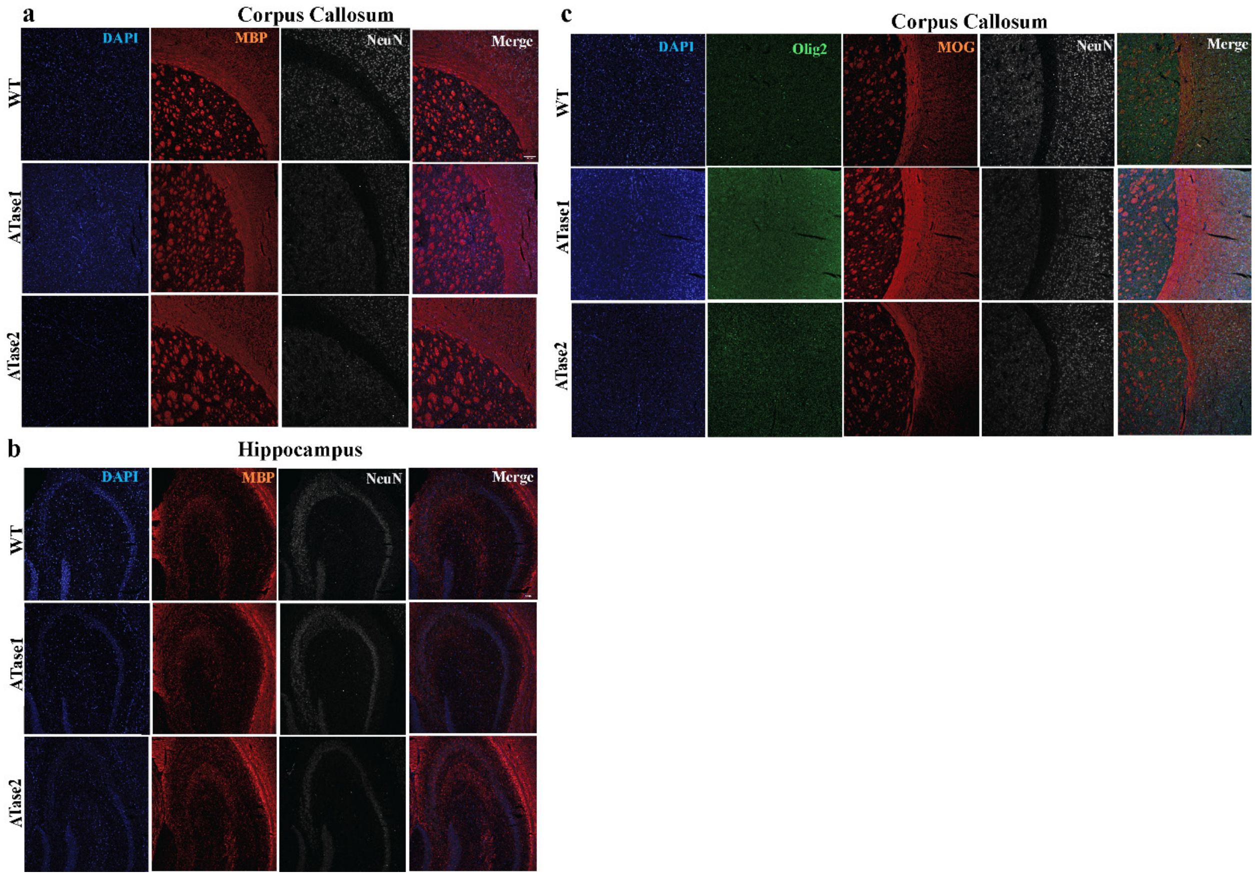

Supplementary Figure 3. ATase1 nTg and ATase2 nTg mice do not demonstrate histologically visible changes in oligodendrocyte abundance. (**a-c**) Paraffin-embedded brain immunostaining. All mice were 7-month-old females.

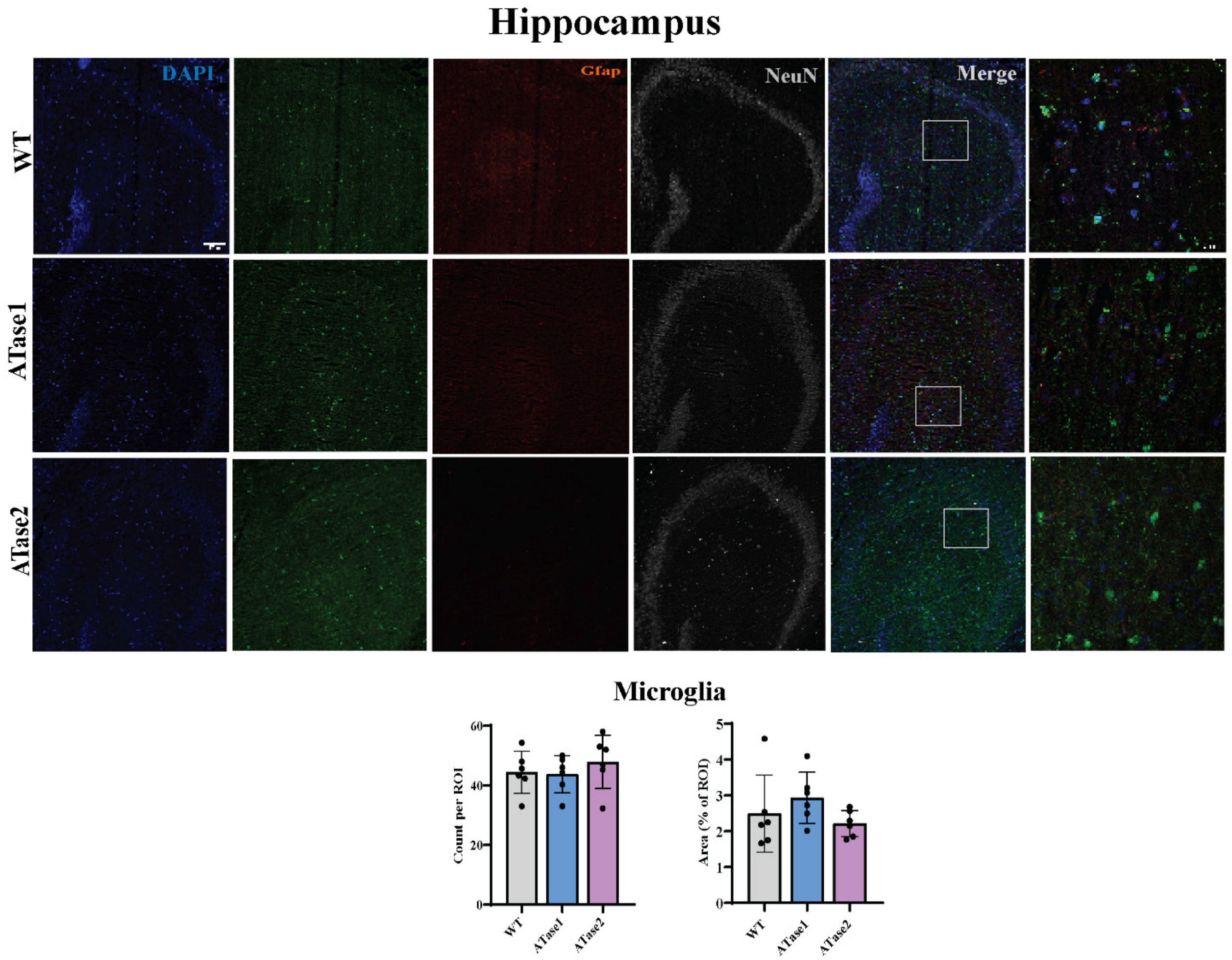

Supplementary Figure 4. ATase1 nTg and ATase2 nTg mice do not demonstrate histologically visible alterations of glial cells. Paraffin embedded brain slices immunostained for microglia (Iba), astrocytes (Gfap) and neuronal marker (NeuN). Iba1-positive cells quantification is shown as mean ± SD. N = 6 mice per genotype (3 males and 3 females) at 7–8 months of age.

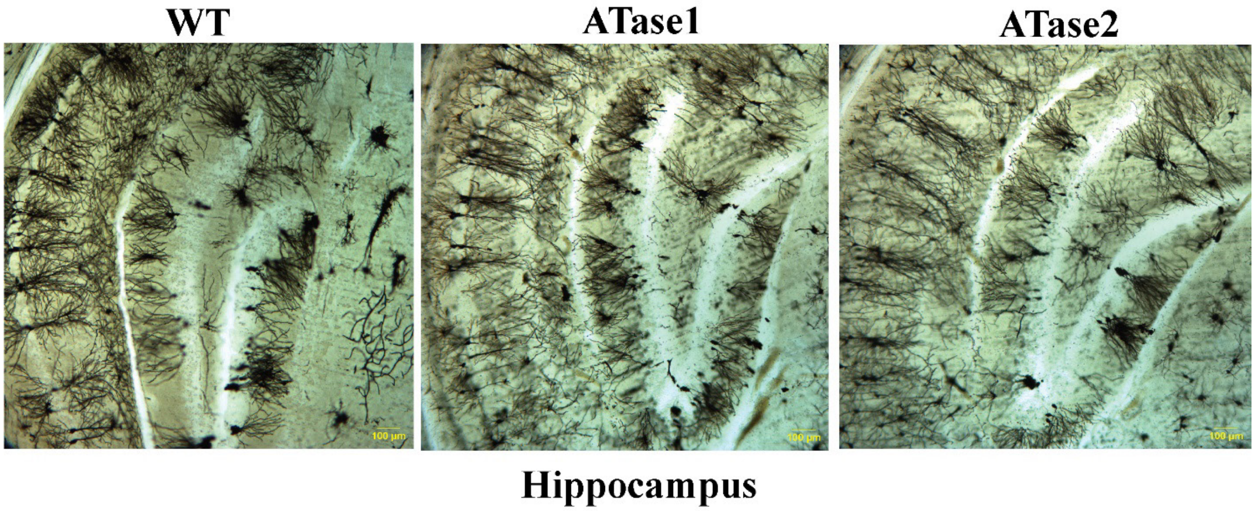

Supplementary Figure 5. ATase1 nTg and ATase2 nTg mice do not display gross alterations with silver/Golgi staining. Mice were 3-month-old males.

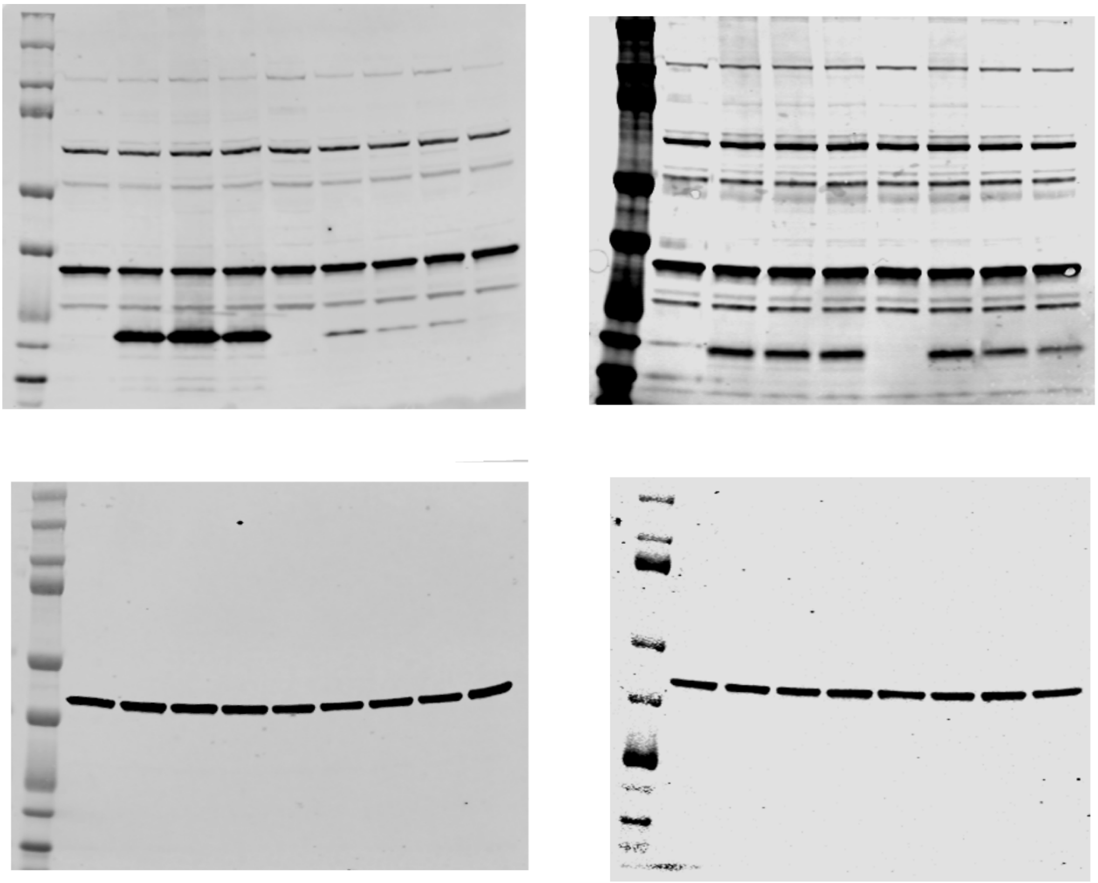

Wb: ATase

Wb: Actin

Supplementary Figure 6. Uncropped Western blots from Figure 1.

Supplementary Movie 1. Typical repetitive jumping phenotype displayed by the nTg mice (only ATase1 nTg is shown).
